# Vitamin C in Plants: From Functions to Biofortification

**DOI:** 10.3390/antiox8110519

**Published:** 2019-10-29

**Authors:** Costantino Paciolla, Stefania Fortunato, Nunzio Dipierro, Annalisa Paradiso, Silvana De Leonardis, Linda Mastropasqua, Maria Concetta de Pinto

**Affiliations:** Department of Biology, University of Bari “Aldo Moro”, Via E. Orabona 4, 70125 Bari, Italy; costantino.paciolla@uniba.it (C.P.); stefania.fortunato@uniba.it (S.F.); nunzio.dipierro@uniba.it (N.D.); annalisa.paradiso@uniba.it (A.P.); silvana.deleonardis@uniba.it (S.D.L.); linda.mastropasqua@uniba.it (L.M.)

**Keywords:** ascorbate, antioxidant, biofortification, light, plant growth, reactive oxygen species, vitamin C

## Abstract

Vitamin C (l-ascorbic acid) is an excellent free radical scavenger, not only for its capability to donate reducing equivalents but also for the relative stability of the derived monodehydroascorbate radical. However, vitamin C is not only an antioxidant, since it is also a cofactor for numerous enzymes involved in plant and human metabolism. In humans, vitamin C takes part in various physiological processes, such as iron absorption, collagen synthesis, immune stimulation, and epigenetic regulation. Due to the functional loss of the gene coding for l-gulonolactone oxidase, humans cannot synthesize vitamin C; thus, they principally utilize plant-based foods for their needs. For this reason, increasing the vitamin C content of crops could have helpful effects on human health. To achieve this objective, exhaustive knowledge of the metabolism and functions of vitamin C in plants is needed. In this review, the multiple roles of vitamin C in plant physiology as well as the regulation of its content, through biosynthetic or recycling pathways, are analyzed. Finally, attention is paid to the strategies that have been used to increase the content of vitamin C in crops, emphasizing not only the improvement of nutritional value of the crops but also the acquisition of plant stress resistance.

## 1. Introduction

Vitamin C (l-ascorbic acid) was isolated from the adrenal cortex by Albert Szent-Györgyi in 1928. Szent-Györgyi demonstrated that this compound, which can act as a powerful reducing agent, indicated with the empirical formula of C_6_H_8_O_6_, had a molecular mass of 178 ± 3 and was a lactone with an acidic hydrogen atom. Due to its similarity to simple sugars and its acidic properties, Szent-Györgyi called this compound “hexuronic acid” [1]. In 1932, Charles Glen King isolated an antiscorbutic compound from lemon juice that was recognized as the hexuronic acid found by Szent-Györgyi [2,3]. At the same time, Szent-Györgyi showed that 1 mg/day of hexuronic acid provided ample protection against scurvy [4]. The definitive structure of vitamin C, which is a hexonic acid aldono-1,4-lactone with an enediol group on C2 and C3, was achieved by Norman Haworth in 1933 [5]. The evidence that this compound was able to prevent scurvy led to it being renamed from hexuronic acid to ascorbic acid [6].

Vitamin C is the most abundant water-soluble compound working in one-electron reactions, and it is an essential micronutrient and a key element for the metabolism of almost all living organisms. In humans, vitamin C has numerous functions, mainly acting as an antioxidant and a cofactor for mono-oxygenases and dioxygenases [7].

The roles of vitamin C as an antioxidant in humans has been established based on a large body of scientific evidence. Vitamin C, by scavenging free radicals, protects DNA, proteins, and lipids from oxidative damages [8]. Vitamin C is used as an antioxidant throughout the body but may have specific roles in some organs. For instance, vitamin C is required in the eyes at a millimolar concentration to guarantee protection from oxidative damage due to solar radiation [9]. Vitamin C inhibits the synthesis of the carcinogenic nitrosamines, which can be synthesized in the intestine or absorbed with food [10], and reduces tetrahydrobiopterin, the cofactor of nitric oxide synthase, which catalyzes the synthesis of nitric oxide [11]. Vitamin C is also important for iron bioavailability, reducing non-heme iron from the ferric (Fe^3+^) to the ferrous (Fe^2+^) form, which is more easily absorbed in the intestine; for this reason, this vitamin is indirectly needed to protect against anemia [12]. Vitamin C also influences iron metabolism through the stimulation of ferritin synthesis and the inhibition of ferritin degradation [13].

Vitamin C, as a cofactor of peptidyl-glycine alpha-amidating monooxygenase, is involved in the biosynthesis of many signaling peptides, such as oxytocin, vasopressin, cholecystokinin, and calcitonin [14,15,16]. Vitamin C functions as a cofactor for many dioxygenases, reducing the iron in the active site of these enzymes to Fe^2+^. Vitamin C contributes to the correct formation of collagen through post-translational modifications of procollagen. In particular, this vitamin acts as a cofactor for the reaction catalyzed by prolyl 3-hydroxylase, prolyl 4-hydroxylase, and lysyl hydroxylase, which are involved in the hydroxylation of lysine and proline and permit the formation of the stable structure of collagen [17,18,19]. Vitamin C, as a cofactor of hydroxylase, is used for the synthesis of norepinephrine and carnitine [20,21,22]. Vitamin C intervenes in many cytochrome-P450-dependent hydroxylation reactions, such as the transformation of cholesterol into bile acids, the degradation of exogenous substances such as pollutants and drugs, and the synthesis of steroid hormones [23].

Recently, vitamin C has been identified as a cofactor for the methylcytosine dioxygenases ten-eleven translocation (TET), which is involved in DNA demethylation and JmjC-domain-containing proteins, which catalyze the demethylation of histones [24]. As a result of vitamin C deficiency, especially in the nucleus, the requirements of TETs or some JmjC-domain-containing histone demethylases may not be met, leading to alterations in the methylation–demethylation dynamics of DNA and histones, which can subsequently contribute to phenotypic alterations or even diseases. By regulating the epigenome, vitamin C can be involved in embryonic development, postnatal development and aging, and cancer and other diseases [24]. Being able to modulate the epigenome, vitamin C has been proposed as an effective molecule in anticancer therapies [25].

Vitamin C is considered a vitamin only for a few vertebrate species, including humans, that are unable to synthesize it [26]. Indeed, vitamin C can be synthesized by plants and most animals [27]. Primates, guinea pigs, bats, some species of birds, insects, invertebrates, and fish are examples of species not able to synthesize this vitamin [26,27]. The inability of humans to synthesize vitamin C lies in the functional loss of the gene coding l-gulonolactone oxidase, the last enzyme involved in the animal biosynthetic pathway of this vitamin [26].

The best way for humans to obtain vitamin C is by diet, and plant foods represent the primary source of this vitamin. Although synthetic vitamin C is chemically indistinguishable from the plant-derived vitamin, fruits and vegetables have different micronutrients and phytochemicals that can affect its bioavailability [28]. Many studies conducted on vitamin-C-deficient animals have shown that vitamin C in plant foods has greater bioavailability than that found in drugs or supplements [28]. For instance, in homozygote Gulo mice, the uptake and tissue distribution of vitamin C were higher when the vitamin was furnished by kiwifruit gel than when it was added as a synthetic supplement in drinking water [29]. Nevertheless, studies conducted on humans have not shown significant differences in bioavailability between synthetic and plant-derived vitamin C [28]. Despite the comparable dosage and bioavailability, it has been shown that orange juice and not synthetic vitamin C drink protects leukocytes from oxidative DNA damage [30]. Probably, the improved effects of vitamin C dispensed with fruits and vegetables is due to the interaction with other micronutrients, such as vitamin E [31] and iron [32]. Consistently, plant-derived vitamin C is related to reduced occurrence of different chronic diseases [33].

The loss of the capability to synthesize vitamin C in our ancestors would not have been a disadvantage with a diet rich in vegetables and fruits, which could have provided enough vitamin C [34]. On the contrary, since l-gulonolactone oxidase produces the potentially toxic H_2_O_2_, this loss could have been an evolutionary improvement in the control of redox homeostasis [35].

Nowadays, very low levels of vitamin C are present in main crops, with the consequence that diet does not provides enough intake of this vitamin. Thus, obtaining plant foods with enhanced vitamin C content represents an important goal for human health. In-depth knowledge of the metabolism and functions of vitamin C in plants is needed to achieve biofortification.

## 2. Vitamin C as an Antioxidant

Vitamin C is an essential element of plant and animal antioxidant systems, which can be defined as complex redox networks, including metabolites and enzymes, with mutual interactions and synergistic effects [36]. Antioxidants can spontaneously provide electrons to free radicals, alleviating the oxidative cellular environments caused by aerobic metabolism.

**Figure 1:**
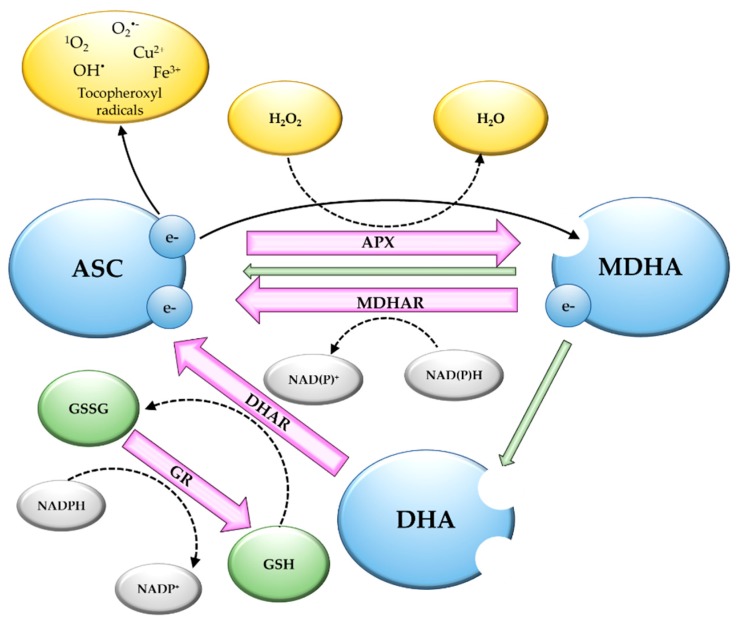
Redox-dependent reactions of vitamin C. ASC can donate electrons directly to reactive oxygen species, metals, and tocopheroxyl radicals. The reduction of H_2_O_2_ by ASC occurs via APX. MDHA can undergo dismutation (green arrows), providing ASC and DHA. The reduction of oxidized forms occurs through the ASC-GSH cycle. MDHA and DHA are reduced by MDHAR and DHAR, respectively, whereas the reduced glutathione is recovered by GR (more details are provided in the text). Abbreviations: ASC, ascorbate; APX, ascorbate peroxidase; DHA, dehydroascorbate; DHAR, dehydroascorbate reductase; MDHA, monodehydroascorbate; MDHAR, monodehydroascorbate reductase; GR, glutathione reductase; GSH, glutathione; GSSG, glutathione disulphide.

Chemically, vitamin C is a dibasic acid with an enediol group on C2 and C3 of a heterocyclic lactone ring. At physiological pH, the hydroxyl group at C3 is deprotonated, giving a monovalent anion, which is indicated as ascorbate (ASC) [37]. The enediol group permits the donation of one or two electrons, forming monodehydroascorbate (MDHA) and dehydroascorbate (DHA), respectively [36]. The ASC redox potential ranges from +0.40 to +0.50 V [38,39]; thus, the molecule can directly donate electrons to reactive oxygen species (ROS), such as singlet oxygen, superoxide anions, and hydroxyl radicals, as well as to tocopheroxyl radicals (Figure 1) [36]. Being able to reduce tocopheroxyl radicals, ASC is indirectly involved in the scavenging of lipid peroxides and radicals, contributing to the decrease of lipid peroxidation and, consequently, to the protection of membranes [40]. Due to the fast reduction of ROS by ASC, the damage of biomolecules can be prevented before the activation of antioxidant enzymes.

ASC can also reduce metals such as copper and iron, leading to the formation of ROS through the Haber-Weiss and Fenton reactions [41]. Thus, in some cases, ASC, acting as a reducing agent, will generate oxidants. This can occur in cell culture media, within the physiologic concentration of ASC, in presence of metals or in vivo in humans only when plasma and extracellular fluids contain millimolar concentrations of ASC [42].

Due to resonance stabilization of unpaired electrons, MDHA, derived from the loss of one electron, has very low reactivity with other radicals and, consequently, has very low toxicity. Two molecules of MDHA can spontaneously dismutate in ASC and DHA (Figure 1) [43]. DHA, if not rapidly reduced to ASC, will be permanently hydrolyzed to threonate, oxalate, oxalyl threonate, or tartrate [44].

ASC has low direct reactivity with H_2_O_2_, but in plants, ASC works as a specific electron donor for ascorbate peroxidase (APX), a heme peroxidase which catalyzes the conversion of H_2_O_2_ to H_2_O and O_2_, giving MDHA (Figure 1). APX has a high affinity for H_2_O_2_ and removes this ROS, even at low concentrations [36]. In plants, different APX isoenzymes have been identified in cytosol, mitochondria, peroxisomes, and chloroplasts, but all are coded by nuclear genes. The plant model system *Arabidopsis thaliana* possesses six APX genes coding for six isoenzymes, two of which are targeted to the cytosol (APX1 and 2), two to peroxisomes (APX3 and 5), one to the thylakoid membrane (tAPX), and one that is dual-targeted to chloroplast stroma and mitochondrial matrix (sAPX) [45]. The crop species rice and tomato possess seven and eight APX isozymes, respectively [46].

APX is part of the ASC-glutathione (GSH) cycle (Figure 1), which is involved in ASC regeneration [47]. MDHA is reduced to ASC by MDHA reductase (MDHAR), which is a flavin enzyme that utilizes NAD(P)H as electron donors [43]. Many cell compartments possess MDHAR activity. *Arabidopsis* has five genes coding for MDHAR2 and 3, localized in the cytosol; MDHAR1 and 4, in peroxisomes and membranes; and MDHAR6 in chloroplasts and mitochondria [48]. DHA can be reduced to ASC by DHA reductase (DHAR), which utilizes GSH as an electron donor, leading to the formation of glutathione disulphide (GSSG). DHAR has an important role in maintaining the reduced ASC in order to avoid DHA degradation [49]. DHAR activity has been identified in cytosol, chloroplasts, mitochondria, and peroxisomes [48]. *Arabidopsis* has three genes coding for DHAR localized differently in the cells: *DHAR1* localized in cytosol and peroxisomes, *DHAR2* localized only in the cytosol, and *DHAR3* targeted to chloroplasts [50]. In the ASC-GSH cycle, GSSG is reduced to GSH by the NADPH-dependent glutathione reductase (GR). GR plays a pivotal role in maintaining the correct balance between reduced GSH and ASC pools [51]. GR activity has been detected in chloroplasts, cytosol, mitochondria, and peroxisomes [52]. In *Arabidopsis*, two genes encode for GR in plants: GR1 is predicted to code a cytosolic isoenzyme and GR2 encodes for a dual-targeted plastidic/mitochondrial protein [53].

Owing to its high antioxidant properties and to the presence of an effective system for redox regeneration, in plants, vitamin C plays a significant role in the defense against oxidative stress, which arises in response to biotic or abiotic stresses [54]. The important role of vitamin C in the tolerance to several stresses is underlined by the increase in the enzymes involved in biosynthesis and recycling, observed in the presence of adverse environmental conditions [49,55]. Interestingly, feeding with l-galactono-1,4-lactone, which enhances the vitamin C content, can increase resistance to various kinds of stress [56,57,58,59].

## 3. Multiple Roles of Vitamin C in Plants

A significant part of accessible glucose (about 1%) is used for vitamin C production, which is present at high concentration in plants [60]. Vitamin C was found in all cell compartments, including the apoplast (the cell wall and extracellular space), reaching a concentration of 20 mM in chloroplasts [61]. However, the vitamin C content significantly differs among plant species and in the same species between diverse cultivars [62]. Moreover, the vitamin C content varies among different tissues and organs, usually being high in leaves, meristematic tissues, flowers, or young fruits and low in non-photosynthetic organs such as stems and roots [54,62]. Only seeds that reach maturity in a stage of strong dehydration (orthodox seeds) contain little vitamin C, which is essentially in the oxidized form [63,64]. In the same organ or tissue, vitamin C content is influenced by the plant developmental stage and environmental changes [65,66,67,68]. Light is one of the most significant environmental signals involved in the regulation of vitamin C levels [69,70].

As in humans, vitamin C favors iron uptake in plants. The ASC efflux in the apoplast contributes to the reduction of Fe^3+^, catalyzed by the ferric chelate reductase plasma membrane enzyme. *Arabidopsis* mutants having low vitamin C content (*vtc* mutants) show a decrease in Fe^3+^ reducing capability and a consequent reduction of iron accumulation in the seeds [71].

Vitamin C, having different functions in chloroplasts, is essential for the correct functionality of photosynthesis. Firstly, ASC has a key role in the direct scavenging of ROS and in the removal of H_2_O_2_ through the water-water cycle [72,73]. ASC also participates in the xanthophyll cycle, which is needed to protect photosystem II (PSII) from photoinhibition. In this cycle, ASC is the cofactor of violaxanthin de-epoxidase, which converts violaxanthin in zeaxanthin, the xanthophyll responsible for dissipating excess excitation energy in the light harvesting complexes of PSII [74]. Finally, ASC can donate electrons to both photosystems, especially when they are damaged by stress conditions [75,76]. Changes in vitamin C content significantly modify the expression of genes linked to photosynthesis [77]. The lowering of the ASC content, through the suppression of DHAR expression, leads to the loss of chlorophyll a, the reduction of the RUBISCO large subunit, and a decrease in CO_2_ assimilation [78]. Consistently, vitamin C-deficient *Arabidopsis* mutants enter senescence earlier than wild-type [79]. Thus, vitamin C, by preserving photosynthetic functioning and limiting ROS-mediated damage, slows down leaf senescence [78,79,80,81].

Vitamin C is involved in the synthesis of the plant hormone ethylene, acting as a cofactor of 1-aminocyclopropane-1-carboxylic acid (ACC) oxidase, the enzyme that catalyzes the last biosynthetic step. Indeed, ASC contributes to the ring opening of ACC by supplying the electron to the active site of the enzyme [62,82]. Being a cofactor of dioxygenases, vitamin C could also be involved in the synthesis of abscisic acid and gibberellins, as well as in the catabolism of auxins [62].

A complex interplay between vitamin C and hormone signaling intervenes in different phases of plant growth and development, as well as in plant response to the environment and pathogens [83,84]. In particular, vitamin C involvement in the defense response against pathogens is strictly dependent on pathogen lifestyles [84]. It is known that defense against biotrophic pathogens is mediated by salicylic acid signaling, whereas defense against necrotrophic pathogens is mediated by jasmonic acid and ethylene signaling [85]. *Arabidopsis* mutants with low vitamin C levels show an increase in salicylic acid, pathogenesis-related proteins, and camalexin and are more resistant to *Pseudomonas syringae* and *Peronospora parasitica* [79,86,87,88]. On the contrary, the same mutants are more susceptible to the necrotrophic ascomycete *Alternaria brassicicola* [89]. Nevertheless, exogenous addition of ASC acts as an inducer of disease resistance in different plant-pathogen interactions [89,90,91,92].

In plants, vitamin C can control the division, elongation, and differentiation of cells, as well as programmed cell death (PCD). Vitamin C plays a significant role in the control of cell division. This metabolite in the meristematic cells of root meristems can shorten the G1 phase and stimulate entry into the S phase [93,94]. In the quiescent center of the root meristem, where cells are not dividing, low levels of ASC, linked to a significant increase of the ASC-consuming enzyme ASC oxidase (AOX), are responsible for the arrest of the cell cycle in the G1 phase [95]. In tobacco BY-2 cells, a peak in ASC, as well as l-galactono-1,4-lactone dehydrogenase (GLDH), activity overlaps with the peak in the mitotic index [96,97]. Moreover, cells enriched with ASC show stimulation of cell division, whereas enrichment with DHA leads to a reduction in cell division, suggesting that the ASC redox state is fundamental to cell cycle progression [96]. Low ASC levels and an altered redox state negatively affect cell cycle progression in the root meristem of *Arabidopsis*, with a consequent decrease in the number of cells in the proliferation zone [98]. An ASC increase has also been shown during cell divisions in developing embryos [99]. The stimulation of division in the apical meristem by ASC seems to be principally due to the inhibition of peroxidase involved in the crosslink of cell wall components. Inhibition of vitamin C biosynthesis leads to abortion of the meristem [100].

The stimulation of cell elongation is due to the expression of AOX, the activity of which determines an increase in oxidized forms of vitamin C in the apoplast [101,102]. Indeed, apoplastic MDHA participates in transmembrane electron transfer, accepting electrons from cytochrome b. This process induces plasma membrane hyperpolarization and activation of H^+^-ATPase, with an acidification of the apoplast that favors cell wall relaxing [103]. The parallel oxidation of NADH acidifies the cytoplasm and activates vacuolar H^+^-ATPase, increasing vacuolization and cell expansion [103,104,105]. In the apoplast, ASC in the presence of Cu^2+^ can exert its pro-oxidant role, producing H_2_O_2_, which induces degradation of polysaccharides [106,107]. Moreover, by reducing lignin precursors utilized by peroxidases, ASC delays cell wall lignification [108]. With the transition from meristematic to differentiated cells, ASC levels significantly decrease, permitting the activity of secretory peroxidases and, consequently, cell wall stiffening and lignification, occurring during differentiation [109].

Vitamin C is also involved in the control of PCD. *A. thaliana* mutants, with low vitamin C content, spontaneously trigger localized cell death like that which occurs during hypersensitive response, a plant-defense mechanism activated to block pathogen invasion [87]. An ASC decrease is necessary for PCD induced by H_2_O_2_ and heat shock (HS) in tobacco BY-2 cells [110,111,112,113]. The decrease in ASC during HS-induced PCD is due to inactivation of the last enzyme of the vitamin C biosynthetic pathway [114]. Low levels of ASC during PCD are parallel to a decrease in the level of transcript, protein, and activity of APX [111,112,115]. The impairment in ASC and APX is needed to increase ROS, which are essential for PCD induction [116,117]. Interestingly, the increase in vitamin C biosynthesis by the supply of l-galactono-1,4-lactone delays PCD occurring during kernel maturation in durum wheat, with a consequent postponement of dehydration and improvement in kernel filling [118].

Vitamin C, regulating the abovementioned processes at molecular and cellular levels, is therefore involved in different phases of plant growth and development, such as seed maturation and germination, flowering, fruit ripening, and senescence [119].

## 4. Vitamin C Biosynthesis in Plants

**Figure 2:**
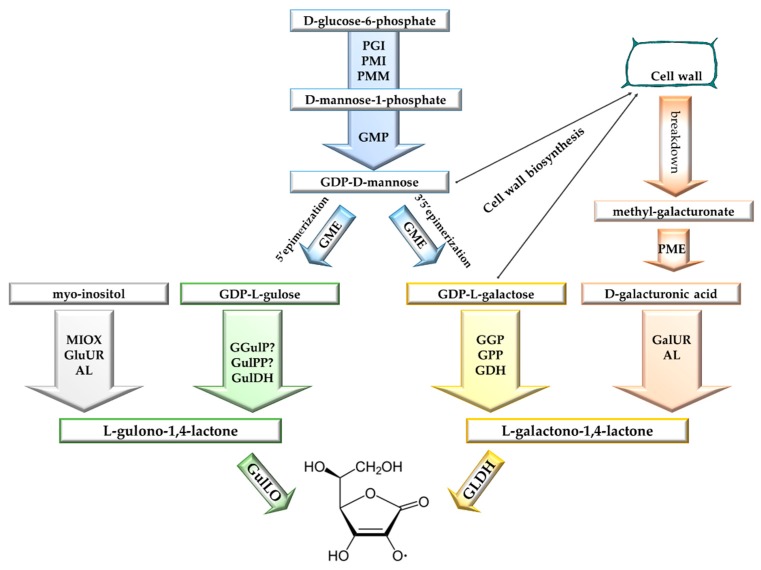
Schematic representation of vitamin C biosynthetic pathways. Different colors indicate different pathways. In grey, the myoinositol pathway; in green, the gulose pathway; in yellow, the d-mannose/l-galactose pathway; in orange, the galacturonate pathway. Represented in blue are the initial steps leading to GDP-d-mannose, which is a common precursor to the d-mannose/l-galactose and gulose pathways. A question mark indicates enzymes not identified in plants (more details are provided in the text). Abbreviations: AL, aldono lactonase; GalUR, d-galacturonate reductase; GDH, l-galactose dehydrogenase; GGP, GDP-l-galactose-phosphorylase; GGulP, GDP-l-gulose pyrophosphatase; GLDH, l-galactono-1,4-lactone dehydrogenase; GluUR, glucuronate reductase; GME, GDP-d-mannose epimerase; GMP, GDP-d-mannose pyrophosphorylase; GPP, l-galactose-1-phosphate phosphatase; GulDH, l-gulose dehydrogenase; GulPP, l-gulose-1-phosphate phosphatase; GulLO, gulonolactone oxidase; MIOX, myoinositol oxygenase; PGI, phosphoglucose isomerase; PME, pectin methyl esterase; PMI, phosphomannose isomerase; PMM, phosphomannose mutase.

Vitamin C biosynthesis in higher plants has been the subject of dispute for many years. The first investigations related to vitamin C biosynthesis in plants date back to 1950 [120]. A definitive mechanism was formulated only 40 years later [60]. Unlike the animal pathway, in plants, no carbon inversion occurs in the biosynthesis of vitamin C, and the C1 in the d-glucose molecule remains as C1 after conversion. The Smirnoff–Wheeler pathway, in which vitamin C is synthesized from d-mannose and l-galactose (d-mannose/l-galactose pathway), represents the major route of vitamin C biosynthesis in plants [62,121]. Three other routes have been proposed for vitamin C biosynthesis: the gulose pathway, the myoinositol pathway, and the galacturonate pathway (Figure 2) [122,123,124].

### 4.1. d-Mannose/l-Galactose Pathway

In the Smirnoff-Wheeler pathway, d-glucose-6-phosphate is transformed into d-fructose-6-phosphate by phosphoglucose isomerase (PGI) and then is directed into d-mannose metabolism by phosphomannose isomerase (PMI), which produces d-mannose-6-phosphate. In *Arabidopsis*, PMI1 expression increases concomitantly with vitamin C levels under continuous light, and knockdown *pmi1* plants showed decreased levels of this metabolite [125]. d-Mannose-6-phosphate is then converted into d-mannose-1-phosphate by phosphomannose mutase (PMM). Genetic evidence for the involvement of PMM in vitamin C biosynthesis has been obtained in *Nicotiana benthamiana* and *Arabidopsis* [126,127].

GDP-d-mannose pyrophosphorylase (GMP) transfers guanosine monophosphate from GTP to give GDP-d-mannose. In *Arabidopsis*, GMP is coded by VTC1; the *vtc1* mutants accumulate ~25–30% of vitamin C levels of wild type and are hypersensitive to ozone [128,129]. *vtc1* mutants also have altered sensitivity to other ROS-generating conditions, including H_2_O_2_, UV-B, SO_2_, and combined high light and salt stress [61]. Additional support for the involvement of GMP in vitamin C biosynthesis was obtained in potato plants constitutively expressing the antisense GMP gene. These plants showed a significant decrease in the activity of the enzyme and a significant reduction of vitamin C in leaves and tubers [130].

GDP-l-galactose is produced directly by GDP-d-mannose through a 3′5′ epimerization catalyzed by GDP-d-mannose epimerase (GME). GME has been characterized in *Chlorella*, flax, and *Arabidopsis* [131,132]. The enzyme belongs to the extended short-chain dehydratase/reductase protein family, with a modified NAD^+^ binding Rossman fold domain [133]. GME is also able to catalyze the 5′ epimerization of GDP-mannose, giving GDP-l-gulose, which is the precursor of a possible side-branch biosynthetic pathway (the gulose pathway) for vitamin C synthesis [122,132,134].

GDP-d-mannose and GDP-l-galactose are substrates for the synthesis of glycoproteins and polysaccharides of cell walls [135,136]. Thus, the first dedicated step for vitamin C synthesis in the d-mannose/l-galactose pathway is the conversion of GDP-l-galactose into l-galactose-1-phosphate, catalyzed by GDP-l-galactose-phosphorylase (GGP) [137].

In *Arabidopsis*, GGP is encoded by the VTC2 and VTC5 genes [121]. The VTC2 expression levels are significantly higher (100–1000 times) than that of VTC5; moreover, T-DNA insertion mutants of VTC2 and VTC5 have 20% and 80% of the vitamin C content of wild-type plants, respectively. The double *vtc2 vtc5* mutants are unable to grow after cotyledon expansion, unless there is feeding of galactose or ASC, suggesting that at least in *Arabidopsis*, the d-mannose/l-galactose pathway is the substantial font of vitamin C [121]. The key role of GGP as a control point in vitamin C biosynthesis has been shown not only in *Arabidopsis* but also in tobacco, tomato, kiwifruit, strawberry, potato, citrus, and blueberry [138,139,140,141,142,143]. The transcript levels of VTC2 and VTC5 strongly correlate with the vitamin C content and the increase with light irradiation [121,144]. Moreover, VTC2, as well as other orthologue genes, can be controlled at the translation level by a noncanonical upstream open reading frame (uORF). In the presence of a high amount of ASC, the uORF encodes for a peptide, which acts as an inhibitor of translation, whereas under a low amount of ASC, the uORF is bypassed and GGP is translated [145].

l-Galactose-1-phosphate is converted into l-galactose by l-galactose-1-phosphate phosphatase (GPP), which is encoded by VTC4 in *Arabidopsis* [146,147]. However, *vtc4* mutants have a partial decrease of GPP activity and vitamin C content [147,148]. Accordingly, in *Arabidopsis*, the reaction can also be catalyzed by the purple acid phosphatase AtPAP15 [149]. l-galactose dehydrogenase (GDH) is the NAD-dependent enzyme catalyzing the conversion of l-galactose into l-galactono-1,4-lactone [60]. This step of ASC biosynthesis is not limiting. Indeed, in *Arabidopsis*, the overexpression of GDH does not change the vitamin C content, and antisense plants show vitamin C decrease only under high light [150].

**Figure 3:**
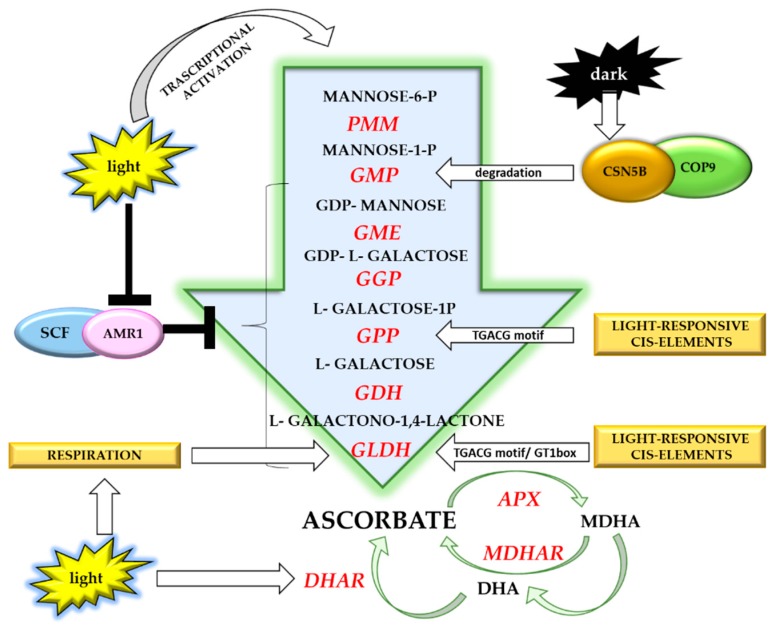
Light-dependent mechanisms involved in vitamin C accumulation (details are provided in the text). Abbreviations: PMM, phosphomannose mutase; GMP, GDP-d-mannose pyrophosphorylase; GME, GDP-d-mannose epimerase; GGP, GDP-l-galactose-phosphorylase; GPP, l-galactose-1-phosphate phosphatase; GDH, l-galactose dehydrogenase; GLDH, l-galactono-1,4-lactone dehydrogenase; APX, ascorbate peroxidase; MDHA, monodehydroascorbate; MDHAR monodehydroascorbate reductase; DHA, dehydroascorbate; DHAR, dehydroascorbate reductase.

The last step of the Smirnoff-Wheeler pathway is catalyzed by GLDH, a flavoprotein which converts l-galactono-1,4-lactone into l-ascorbate, transferring electrons to cytochrome c [27,120,151]. An important observation is that GLDH does not release H_2_O_2_, as happens with l-gulonolactone oxidase in animals, and therefore the production of vitamin C in plants does not affect the redox state of the cell [152]. Unlike the other enzymes of the d-mannose/l-galactose pathway, which are all localized in the cytosol, GLDH is an integral protein of the mitochondrial inner membrane [97,153,154]. Specifically, GLDH has been detected in complex I and acts as an essential plant-specific factor for complex I assembly [155,156,157,158]. Due to the GLDH localization, vitamin C biosynthesis is very sensitive to stresses that cause impairments of electron flux [111,114,159]. On the other hand, an increase in respiration, like that observed during tomato ripening, is associated with an enhancement of vitamin C content [160]. The different steps of the Smirnoff-Wheeler pathway are schematized in Figure 2 and Figure 3.

### 4.2. Other Vitamin C Biosynthetic Pathways

As reported above, the gulose pathway starts from the 3′ epimerization of the GDP-d-mannose catalyzed by GME, with the formation of GDP-l-gulose (Figure 2) [122]. In this pathway, GDP-l-gulose is successively converted into l-guolse-1P, l-gulose, and l-gulono-1,4-lactone. l-gulono-1,4-lactone has been detected in plant extracts [161]. Moreover, external supplementation of l-gulono-1,4-lactone causes an increase in vitamin C content in *Arabidopsis* cells and tobacco leaves but is less efficient than feeding with l-galactono-1,4-lactone [162,163,164]. Activity of gulonolactone oxidase (GulLO) has been found in potato [163], and more recently, two genes coding for GulLOs have been identified in *Arabidopsis*. These enzymes are dehydrogenases with specificity for l-gulono-1,4-lactone and differ from plant GLDHs and mammalian GulLOs. The *Arabidopsis* GulLOs seem to be regulated post-transcriptionally and the limited enzyme availability could explain the slow utilization of the substrate [165].

l-Gulono-1,4-lactone is also the last precursor of vitamin C biosynthesis in the myoinositol pathway (Figure 2). In this pathway, myoinositol is converted into d-glucuronate by myoinositol oxygenase (MIOX). The other two steps producing l-gulonic acid and l-gulono-1,4-lactone are respectively catalyzed by glucuronate reductase and aldono lactonase [166]. A myoinositol oxygenase (MIOX4) has been identified in *Arabidopsis* [123]. However, the effective contribution of myoinositol to vitamin C synthesis in vivo is strongly debated [167,168,169].

The galacturonate pathway is also known as the salvage pathway, since it utilizes sugars provided by the breakdown of cell walls [124]. The degradation of pectin releases methyl-galacturonate, which is converted into d-galacturonate by methyl esterase and successively into l-galactonate by d-galacturonate reductase (GalUR). An aldono lactonase converts l-galactonate into l-galactono-1,4-lactone, which is the last precursor of vitamin C in the Smirnoff–Wheeler pathway (Figure 2) [27]. A gene encoding GalUR was initially identified in strawberry [124,170]. This pathway seems to be active mainly during fruit ripening in some species [124,170,171,172,173]. In tomato, vitamin C synthesis in immature green fruit is enhanced only by the supply of l-galactose, whereas in red ripened fruits by feeding with both l-galactose and d-galacturonate [170]. Moreover, the high vitamin C content found in tomato introgression lines IL12–4 compared with the parental M82 seems to be due to a higher expression of a pectinesterase and two polygalacturonases [171].

## 5. Light-Dependent Vitamin C Accumulation in Plants

Several papers have reported that plant exposure to light significantly increases vitamin C content [139,174,175]. Consistently, probably due to a reduction of irradiance levels, plants grown in greenhouses show lower levels of vitamin C compared with plants cultivated in the field [176]. However, the enhancement of vitamin C in plants seems to be dependent on the total amount of incident global radiation, which can be regulated by modulating light intensity or the duration of plant’s exposure to light. The use of continuous light for 48 and 72 h after a period of darkness causes a great increase of vitamin C levels in lettuce and *Arabidopsis*, respectively [70,177]. However, the continuous exposure of lettuce plants to very high irradiation causes a loss of vitamin C, whereas continuous low irradiation improves the content of this metabolite [178]. Shen et al. [179] found that in lettuce, continuous illumination with red-blue light emitting diodes (LEDs) increases the vitamin C content in relation to the exposure time. Interestingly, in the same plants cultivated for 15 days under continuous red-blue LEDs exposure, the maximum peak of vitamin C content was found after nine days from the beginning of light exposure [180].

The quality of light can also influence the vitamin C pool. The regulatory effects of monochromatic lights of the UV-Vis spectrum on the modulation of vitamin C content have been studied in different species, but the obtained results are often contradictory, suggesting that different species can respond differently to specific wavelengths. High-red/far-red ratios enhanced vitamin C levels in the leaves of *Phaseolus vulgaris* [67]. Lettuce plants illuminated with single or combined blue and red lights showed higher vitamin C contents than plants grown in white light [181]. In Chinese kale, different lights, except for blue, used during sprout growth improved the vitamin C content, and the highest concentration of ASC was found in shoots exposed to white LEDs [182]. The influence of light on the vitamin C content was also evaluated in numerous fruits, such as apple [183], tomato [175,184], Satsuma mandarin, Valencia orange, and Lisbon lemon [185]. In these three citrus varieties, the enhancement of vitamin C content in the fruits was greater with the increasing intensity of blue LEDs. Furthermore, continuous irradiation with blue LEDs is more effective than pulsed irradiation and is related to increased expression of genes involved in the modulation of the vitamin C pool, suggesting a control at the transcriptional level [185]. On the contrary, accumulation of vitamin C in lettuce grown under continuous light is mainly due to changes in activity, rather than in expression, of the enzymes involved in vitamin C biosynthesis and oxidation [180].

Light treatments have also been tested in the postharvest, but also in this case, contradictory results have been reported. In cabbages stored for 15 days at low temperatures, the content of vitamin C was higher in the presence of blue light [186], unlike what was observed in asparagus stored at 4 °C, in which the vitamin C content after six days of blue light did not differ from the control in the dark [187]. In broccoli as well, blue light had no positive effects on the vitamin C content, whereas green, red, and yellow lights, probably stimulating metabolic and physiological activity, permitted de novo vitamin C synthesis [188].

In addition to visible light radiation, plants are also exposed in nature to UV radiation, which constitutes about 7% of solar radiation [189]. UV was shown to improve vitamin C content in soybean sprouts [190]. Increased levels of vitamin C in cucumber plants illuminated with UV-B have been linked to a significant increase in the activity of MIOX, GLDH, and enzymes of the ASC-GSH cycle [191].

### Light Regulation of Vitamin C Accumulation

The pivotal role of light-dependent vitamin C accumulation in green tissues is due to photosynthesis. In *Arabidopsis* leaves and tomato green fruits, the photosynthetic inhibitor DCMU blocks vitamin C accumulation by reducing the expression of genes involved in its biosynthesis [70,170]. Moreover, photosynthesis increases the amount of soluble carbohydrates, which are biosynthetic precursors of vitamin C. Three genes involved in carbohydrate accumulation and translocation have been related to a quantitative trait locus (QTL) associated with a 1.4-fold vitamin C increase in tomato [192]. On the other hand, Ntagkas et al. [193] found no correlation between vitamin C content and levels of soluble carbohydrates.

Light can control vitamin C accumulation through different types of regulation (Figure 3).

Light-dependent accumulation of vitamin C in plants seems to be principally due to the enhanced expression of different genes involved in the d-mannose/l-galactose biosynthetic pathway [70,121,142,175]. It has been shown that in rice, GLDH and GPP contain light-responsive cis-elements (GT1 box and TGACG motif) in their promoters [194]. Concerning GLDH, light beyond regulating its expression can induce changes in respiration, indirectly modulating enzymatic activity [69,159]. Light also influences the expression of genes involved in ASC recycling. During germination, corn seeds exposed to high light exhibited a higher DHAR expression along with an increased vitamin C content (Figure 3) [195].

In tomato, light induces the expression of the transcription factor HZ24, which in turn activates GMP transcription in leaves and immature fruits [196]. High light also induces a decrease in the expression of AMR1, a transcription factor acting as a negative regulator of the last six genes of the d-mannose/l-galactose biosynthetic pathway, consequently increasing vitamin C biosynthesis (Figure 3) [197]. Finally, in the dark, CSN5B promotes GMP degradation via 26S proteasome, reducing vitamin C levels [198]. Additionally, darkness promotes vitamin C catabolism [44].

The complexity of light-dependent vitamin C regulation (Figure 3) highlights that obtaining vitamin-C-biofortified plants by light treatments requires in-depth knowledge of the metabolic and physiological processes involved [199].

## 6. Vitamin C Biofortification

**Figure 4:**
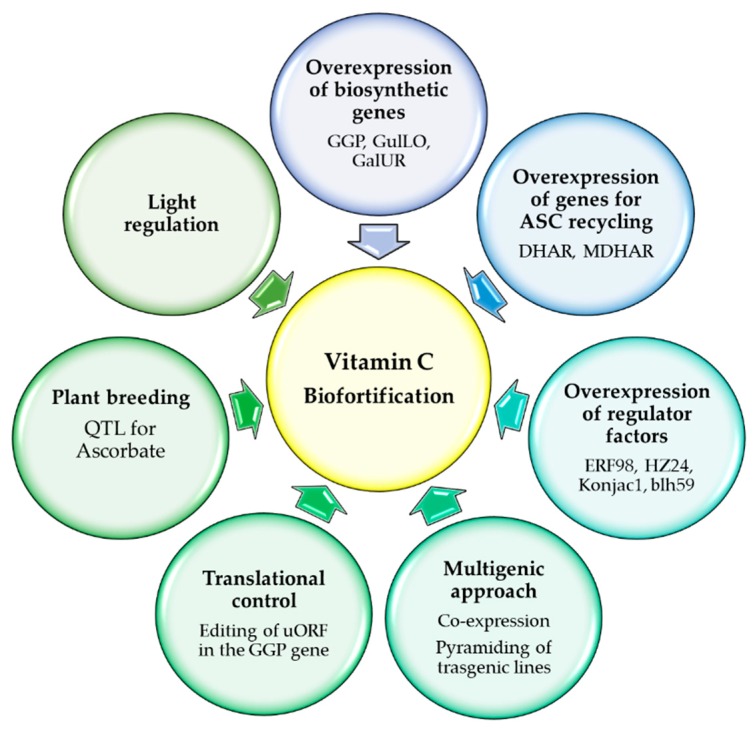
Strategies for vitamin C biofortification. Modulation of light intensity and quality can be used to obtain vitamin C enrichment in crops. The overexpression of single or multiple genes belonging to the biosynthetic and recycling pathways, as well as to the regulatory network, represents a good tool for vitamin C biofortification. Vitamin C accumulation can be controlled by modulating translation by the editing of the uORF in the GGP gene. Finally, vitamin C biofortification can be obtained by plant breeding that exploits candidate genes in the quantitative trait locus (QTL) associated with high vitamin C content (more details are provided in the text).

Increasing the vitamin C content in plants can have a triple-positive effect: producing food with a high content of vitamin C for human health, increasing the postharvest shelf life, and, not less important, increasing the resistance of plants to various kinds of stress. The different strategies adopted for vitamin C biofortification (Figure 4) are discussed below.

### 6.1. Manipulation of d-Mannose/l-Galactose Pathway

Several genes of the d-mannose/l-galactose pathway have been overexpressed in different crops in order to enhance vitamin C levels, but not all have given good results [34,35]. It has been proved that overexpression of GGP, which represents the bottleneck of vitamin C biosynthesis [137], is a good strategy for biofortification [200]. For instance, in *Arabidopsis*, transient overexpression of GGP leads to a 2.5-fold increase in vitamin C content, whereas the overexpression of the other genes involved in the same pathway does not cause relevant differences in terms of vitamin C [142]. Similar results were obtained in rice where, among different transgenic lines overexpressing six *Arabidopsis* genes involved in vitamin C biosynthesis, the highest vitamin C content was found in the line overexpressing GGP [201]. A kiwi gene coding for GGP, initially tested with good results in *Arabidopsis* [138], has been overexpressed in three crops, leading to a vitamin C increase of six-fold in tomato, three-fold in potato, and two-fold in strawberry; however, in tomato, GGP overexpression has led to some morphological fruit alterations, such as seed loss [140]. The GGP gene of acerola, a well-known crop with high vitamin C content, under the control of a leaf-specific promoter, has been overexpressed in rice, increasing the foliar content up to 2.5-fold, which did not cause morphological changes and conferred multistress tolerance [202]. Interestingly, also, the editing of the uORF, which controls the translation of the GGP2 gene in lettuce and tomato, increases the vitamin C content by 150% and confers tolerance to oxidative stress, providing a good strategy to obtain transgene-free lines with improved vitamin C [203,204]. Moreover, in apple, three paralogs of GGP, colocated in ASC-QTL clusters and, specifically, the GGP1 allele, play a key role in the regulation of vitamin C content in fruits. This suggests that a single-nucleotide polymorphism of this allele is an excellent candidate for breeding in order to improve vitamin C levels in fruits [205].

The multigenic approach, based on the coexpression of genes of the d-mannose/l-galactose pathway, represents an interesting strategy to obtain high levels of vitamin C in crops. The transient coexpression of GGP and GME in tobacco leaves caused a seven-fold increase in vitamin C content [138]. In *Arabidopsis*, GGP overexpressing lines had a 2.9-fold enhancement of vitamin C, whereas the double-gene transformation with GGP-GPP and GGP-GLDH led to an up to 4.1-fold vitamin C increase [206]. The contemporary overexpression of acerola GGP, GMP, and GME genes in tomato protoplasts caused an increase in vitamin C content, which was approximately four-fold higher than in wild type [207]. A stable transformation with GME, GMP, GGP, and GPP was obtained in tomato through pyramiding, which is a conventional hybridization that is technically achievable and generates stable inherited target genes [208,209]. Pyramiding transgenic lines GME × GMP and GME × GMP × GGP × GPP showed a substantial increase in vitamin C content in leaves and fruits. Moreover, in these lines, vitamin C transport capability, fruit shape and size, as well as stress tolerance were significantly ameliorated [209].

### 6.2. Manipulation of Other Biosynthetic Pathways

The overexpression of genes of the alternative biosynthetic pathways have also given good results in terms of vitamin C content in different crops. Regarding the gulose pathway, positive results have been obtained with the expression of rat cDNA encoding GulLO, the enzyme involved in the final step of the animal vitamin C biosynthetic pathway [163,210,211]. Lettuce and tobacco plants constitutively expressing this gene showed four- and seven-fold increases in vitamin C levels, respectively [163]. Transgenic potato plants, overexpressing the same gene, show improved vitamin C accumulation in tubers and increased tolerance to several abiotic stresses [210]. In the same way, *Arabidopsis* lines overexpressing this GulLO contained high vitamin C contents and exhibited improved growth and enhanced biomass of shoots and roots, as well as higher tolerance to diverse abiotic stresses [211].

Interesting results have also been obtained by manipulating the galacturonate pathway. Overexpression of the strawberry FaGalUR led to a two-fold increase in vitamin C content in potato, and this enhancement allowed for an increase in tolerance to abiotic stresses in the transgenic lines [212]. Similar positive results have been reported for tomato, where, although there was a moderate increase in vitamin C content, an increase in total antioxidants occurred that was linked to redox state regulation [213]; moreover, tomato plants overexpressing FaGalUR were found to be more tolerant to abiotic stresses [214]. Interestingly, in the tomato introgression line IL12-4-SL, the genes encoding for pectin methylesterase, polygalacturonase, and UDP-d-glucuronic-acid-4-epimerase, which are involved in pectin degradation, have been identified as candidate genes for a QTL associated with high vitamin C content, suggesting that marker-assisted selection could be a good strategy to enhance vitamin C accumulation [215].

### 6.3. Manipulation of Recycling Genes

Vitamin C enhancement in crops can also be achieved by manipulating the genes coding for MDHAR and DHAR, which are the enzymes involved in the reduction of MDHA and DHA, respectively. Several papers have reported that ASC regeneration by DHAR overexpression could represent an efficient method of vitamin C biofortification in different species, such as corn [216], tomato [217], and blueberry [143]. Recently, a cytosolic DHAR identified in the woody plant *Liriodendron chinense* was overexpressed in *Arabidopsis*, which led not only to vitamin C enhancement but also to an improvement of growth under stress conditions [218]. In apple, colocation between *DHAR3-3* and a QTL for browning has been found, showing a relationship between ASC redox state and fruit vulnerability to browning [205].

Research conducted on MDHAR shows discordant results depending on the species. The overexpression in tobacco of the *Arabidopsis* cytosolic isoform of MDHAR enhances vitamin C content [219]. Similarly, the acerola MDHAR, overexpressed in tobacco, led to a two-fold increase in vitamin C content and a better tolerance to salt stress [220]. On the other hand, overexpression of the cytosolic-targeted tomato MDHAR caused a 0.7-fold reduction in vitamin C content in tomato fruits [217]. Another study conducted on tomato indicates that transgenic lines overexpressing MDHAR display a reduction in vitamin C content in leaves, while lines with silencing of MDHAR show an increase of vitamin C in both fruits and leaves [221]. The enhancement of vitamin C in silenced MDHAR lines could be due to a decrease in degradation [44]. In cherry tomato, the suppression of AOX was also found to increase vitamin C, lycopene, and carotene contents of the fruits and to confer tolerance to salt stress [222].

### 6.4. Manipulation of Regulatory Networks

Despite the many results obtained by overexpressing vitamin C-related genes, limited success has been reported in most species. In light of this, attention has shifted to the manipulation of components of the regulatory network, such as transcription and regulator factors. The overexpression of ERF98, which is a positive regulator of GMP, GGP, and GLDH genes, enhanced vitamin C content and increased tolerance to salt stress in *Arabidopsis* [223]. Similarly, tomato plants overexpressing HZ24, a transcriptional factor that binds the promoters of GMP, GME2, and GGP, showed increased vitamin C levels and reduced sensitivity to oxidative stress [196]. Overexpression of the regulator factors KONJAC1 and 2, which are two nucleotide sugar pyrophosphorylase-like proteins that modulate GMP activity, led to an increase in vitamin C content in *Arabidopsis* [224]. *Arabidopsis* and tomato plants, overexpressing the regulator factor SlZF3, showed inhibition of GMP degradation by COP9 signalosome, with a consequent enhancement of vitamin C content and tolerance to salt stress [225]. A new transcription factor bHLH59, which can activate the transcription of PMI, PMM, and GMP 2–4 and colocalize with the vitamin C QTL TFA9, has been identified in tomato. The overexpression of bHLH59 causes vitamin C accumulation and increases oxidative stress tolerance. The differences in vitamin C accumulation within different tomato accessions is ascribed to nucleotide differences in the promoter region of HLH59. This finding could be used to plan breeding strategies for vitamin C improvement [226].

## 7. Conclusions

In humans, different physiological processes require vitamin C as an antioxidant or a cofactor of mono-oxygenases and dioxygenases, whereby low vitamin C levels prevent optimal functioning. An increase in vitamin C intake through food is surely beneficial for human physiology [227,228]. The recommended daily intake (RDI) of vitamin C is 75–90 mg/day [229]. Nevertheless, 100 g of potatoes and tomatoes have about one-fourth of the RDI and cereal grains contain very low, almost undetectable, quantities of vitamin C [230]. Assuming their potential to make available adequate vitamin C levels, biofortified crops could be decisive in the elimination of vitamin C deficiency on a worldwide scale. Apart from having beneficial effects on human health, vitamin C biofortification also has the potential to improve plant tolerance to various stresses, which is a prominent target to guarantee crop productivity in an era of global climate change.

However, vitamin C accumulation in different plant organs is dependent on multiple metabolic processes, such as biosynthesis, recycling, degradation, and transport. Moreover, the vitamin C content is influenced by endogenous stimuli and environmental factors, among which light is of primary importance. Thus, as for other micronutrients, comprehensive knowledge of the genetic, biochemical, and molecular networks that govern vitamin C levels is mandatory to obtain vitamin-C-biofortified crops [231].

Considerable progresses on the understanding of the multiple roles of vitamin C and its interaction with other antioxidants, as well as with signal transduction pathways of hormones and ROS have been made. However, little is known about the influence that increased vitamin C levels may have on the different physiological processes of plants. Since changes in vitamin C levels greatly modify gene expression, and in particular, the transcript levels of genes involved in photosynthesis and the defense response to pathogens [77], the possibility of undesirable consequences resulting from the altered vitamin C content has to be carefully considered. Thus, efforts to obtain vitamin-C-biofortified plants necessitate an in-depth investigation into how these changes can alter plant growth, development, and responses to biotic and abiotic stresses under field conditions. To limit the possible unplanned consequences, targeted approaches altering vitamin C levels in specific tissues or organs are required.

Particular attention must be also paid to the choice of methodology utilized for vitamin C biofortification. The multigenic approach, obtained with co-expression or pyramiding, has led to a significant increase in vitamin C content. However, considering that genetically modified organisms are not always easily accepted by public opinion, methodologies avoiding the use of transgenes must be taken in consideration. For instance, the editing of the uORF on the promoter of genes coding for GGP is a good method to obtain non transgenic plants enriched with vitamin C. The identification of candidate genes in QTL associated with high vitamin C content could also allow for obtaining vitamin-C-biofortified plants by marker-assisted selection, thus avoiding the use of transgenes.

Thoroughly understanding the regulatory mechanisms involved in vitamin C accumulation, which can differ between crops and growth phases, together with the choice of better approaches to be utilized to improve vitamin C levels are important goals for developing more efficient strategies for vitamin C biofortification.
